# The Impact of Self-Efficacy Analysis-Based Psychological Theory and Literary Ethics on Chinese American Entrepreneurship Education

**DOI:** 10.3389/fpsyg.2020.01870

**Published:** 2020-08-04

**Authors:** Hang Li, Junsheng Wang, Yunyu Zhang, Hongmei Li, Xialu Chen

**Affiliations:** ^1^School of Foreign Languages, Chongqing Jiaotong University, Chongqing, China; ^2^Graduate School of Pan-Pacific International Studies, Kyung Hee University, Yongin-si, South Korea; ^3^School of Law, Xiamen University, Xiamen, China; ^4^School of Education, Jinggangshan University, Ji’an, China; ^5^Department of Human Resources, Chongqing Vocational Institute of Engineering, Chongqing, China

**Keywords:** entrepreneurial efficacy, psychological state, literary ethics, entrepreneurial intention, entrepreneurship education

## Abstract

In this study, entrepreneurship education was explored from the perspective of the combination of psychology and literary ethics, with the purpose of studying the entrepreneurial behavior of Chinese American college students and promoting the development of entrepreneurship education. Based on the analysis of self-efficacy, the correlations among entrepreneurial intention, entrepreneurship education, and entrepreneurial efficacy of the research samples were analyzed. First, through the questionnaire design, the research samples and the measurement scales of each research variable were determined, and the survey results and the reliability of the scale were analyzed and tested. Second, based on the variance analysis and regression analysis methods, a descriptive statistical analysis was performed on the correlations among entrepreneurship education, entrepreneurial intentions, and entrepreneurial efficacy among Chinese American college students. Finally, the idea of literary ethics was integrated into entrepreneurship education, entrepreneurial intentions, and entrepreneurial self-efficacy, and the correlation structure model was constructed. The intermediary role of entrepreneurial efficacy in entrepreneurship education and entrepreneurial intention was tested. In addition, the individual gender and family entrepreneurial behaviors were considered. The results show that the valid response rate of the questionnaire, is satisfactory at, 96.49%; the reliability and validity of the scales of the research variables are satisfactory; the Cronbach’s Alpha reliability coefficient values are all above 0.80; and the fitting results of the confirmatory factors are satisfactory. The regression analysis results show significant correlations among entrepreneurship education, entrepreneurial intentions, and entrepreneurial efficacy among Chinese American college students. Entrepreneurial efficacy has a partially intermediary role in the two dimensions of entrepreneurship education and entrepreneurial intention. Individual gender and family entrepreneurial behaviors have moderating effects, on the entrepreneurial efficacy levels of college entrepreneurs. From the perspectives of psychology and literary ethics, the above results have positive effects on the development of entrepreneurship education.

## Introduction

With the economic development and the increasingly fierce market competition environment, entrepreneurial activities have attracted wide attention. For college students, entrepreneurship has become a new way to achieve employment (Kheng, 2017; Bogusz and Morisse, 2018). Entrepreneurship education was first developed in developed countries in the 20th century. Some studies have found that more than three-quarters of American colleges and universities set up entrepreneurship courses (Hrisch et al., 2017; Shelton, 2017). For entrepreneurship education, currently, discussions and research have been performed on the promotion of production levels and technological innovation (Izzo, 2017), its correlations with entrepreneurial intentions and opportunity recognition (Allini et al., 2017), and the content of entrepreneurship education (Massis et al., 2017). From the perspective of culture and entrepreneurship, entrepreneurial intentions and motives of entrepreneurs in different environments are different. Especially, in the context of a country with a large environmental differentiation, such differences will be more obvious. The entrepreneurial traits possessed by individuals are closely related to their psychological behaviors (O’Shea et al., 2017). To analyze entrepreneurship education from a psychological perspective, the element of entrepreneurial self-efficacy is an important embodiment of personality psychological traits, both in terms of tasks and fields (Cho et al., 2017). From the perspective of ethics, it is meaningful to analyze and explore the self-employment behavior of college students. However, there are not many research results in this field globally. The main focus of ethics theory is on ethics education (Sergey and William, 2017). Currently, there is little research on entrepreneurship education based on ethics, and there is also little research on the combination of the psychological characteristics and ethics of entrepreneurs.

Therefore, Chinese American college students were included as the research samples. The idea of literary ethics was introduced in an innovative manner. This study aims to explore and analyze the correlations among entrepreneurial self-efficacy, entrepreneurial intention, and entrepreneurial education based on psychological theory. It is hoped that this study may provide some theory and data references for the development of entrepreneurship education based on psychological theory and ethics perspective.

## Literature Review

### International Research Progress

Tingey et al. (2020) studied the impact of entrepreneurship education programs on youth entrepreneurship knowledge and social welfare, and found that related entrepreneurial interventions had a positive impact on the development of youths (Tingey et al., 2020). Suryavanshi et al. (2019) explored the correlation between health care and entrepreneurship and showed that the groups that will play a greater role in the medical industry in the future would mainly focus on innovative entrepreneurs. Mollica et al. (2017) explored the impact of school entrepreneurship and management courses on students’ entrepreneurial self-efficacy and revealed that entrepreneurship and management training were very helpful for students to plan their careers. Asimakopoulos et al. (2019) analyzed and explored the role of entrepreneurship education in entrepreneurial intentions and found that entrepreneurial education had a positive regulatory role in entrepreneurial self-efficacy and entrepreneurial intentions. Christensen et al. (2018) discussed the integration of ethics education into the accounting curriculum, and through comparison with traditional moral education, it was found that the introduction of ethics education is meaningful for the cultivation of students’ satisfactory, moral quality. Ramus and Vaccaro (2017) researched and discussed the positioning and balance between corporate wealth creation and social value creation.

### Research Progress in China

Wan and Wang (2018) studied the role of sellers’ entrepreneurial self-efficacy and remote work self-efficacy in operational creativity in the market and found that the two have a synergistic effect in promoting creativity. Zhang et al. (2019) explored the influence of psychological factors on entrepreneurs’ causal decision-making logic preferences, and the research results show that psychological factors have an influence on the entrepreneur’s causal and effective decision-making logic preferences; samples from the United States and China showed positive correlations existing among self-efficacy, control decision logic, and predictive decision logic.

In summary, works that explore entrepreneurship education, entrepreneurial intentions, psychological factors, and other levels are various. Therefore, entrepreneurship education has attracted the attention of scholars all over the world. Entrepreneurial self-efficacy has a significant influence on entrepreneurship education. Individual psychological factors have a greater influence on the choices of entrepreneurship and development orientation. The role of ethics education in cultivating the satisfactory, quality of students cannot be ignored. However, research that introduces ethical ideas into entrepreneurship education is rare, as well as research that combines academic theory and literary ethics.

## Materials and Methods

### Entrepreneurial Efficacy Based on the Field of Psychology

The concept of self-efficacy was first proposed by the psychologist Bandura. Based on the category of psychological theory, it represents the individual’s prediction, belief, or confidence in the ability required in the process of achieving a given goal (Dai et al., 2019). A lot of studies have shown that individual emotions and their physiological conditions are one of the key factors affecting self-efficacy (Yang et al., 2018). In the field of entrepreneurship education, entrepreneurial self-efficacy is a term defined under the combination of self-efficacy and entrepreneurial management. It can also be referred to as entrepreneurial efficacy. Entrepreneurial efficacy is a manifestation of entrepreneurs’ psychological traits. It has a predictive ability for entrepreneurial behaviors and activities. During interaction with the external environment, entrepreneurial efficacy can be enhanced and improved further. Entrepreneurial efficacy is defined as the perception and belief of individual entrepreneurs of Chinese American college students regarding the entrepreneurial behaviors. With the exploration and measurement of entrepreneurial efficacy and the structure of exploration, the Entrepreneurship Self-Efficacy Scale, which effectively distinguishes entrepreneurs from non-entrepreneurs, and the Entrepreneurial Efficacy Evaluation Scale, which effectively distinguish entrepreneurs and managers and includes six dimensions, are proposed successively. On this basis, some scholars have divided entrepreneurship effectiveness into four dimensions: opportunity identification, correlations, management, and risk-taking (Nakamura, 2019). At present, there is no unified standard for cognition of the dimension of entrepreneurial efficacy. Therefore, multiple dimensions were chosen to measure entrepreneurial efficacy in this study.

### Basic Theory of Literary Ethics

Literary ethics is a discipline that studies the creation, criticism, and reading of literary works, as well as the ethical values and ethics related to literature. This special discipline covers a wide range of content, and it mainly applies knowledge about ethics to solve moral problems in literature. Literary ethics, as the name implies, is a combination of literature and ethics, both of which are related to moral issues; however, the difference is that ethics discuss moral issues in real life, while literary ethics focuses on the discussion and analysis of moral issues in a virtual environment. By integrating ethics into literature, this discipline provides a new direction for the development of literature. At the same time, in combination with literary characteristics, it is also conducive to the development and deepening of ethics in the research process of ethics. The combination of literary ethics and “learning” is more ethical. Entrepreneurship is a very complicated project that involves many interpersonal interactions. The complex ethical and moral issues that entrepreneurs will encounter are endless, such as the formation of entrepreneurial innovation spirit, the improvement of entrepreneurs’ individual comprehensive quality, the establishment of moral personality, the perseverance in belief perception (the improvement and deepening of entrepreneurial efficacy), and entrepreneurial ethical cares from all aspects. The above are all parts of entrepreneurial ethics. Therefore, from the perspective of literary ethics, the impact of entrepreneurship education on the entrepreneurial community of Chinese American college students is explored. From the perspective of entrepreneurship education, compared with traditional ethics, the introduction of literary ethics has improved the comprehensive quality of entrepreneurship for Chinese American college students, the cultivation of the moral quality of entrepreneurial individuals, and the establishment of moral personality. It even plays a role in the improvement of psychological quality during the entrepreneurial process and the successful realization of entrepreneurship. The role of literary ethics in the entrepreneurial process of college students is subtle, which is shown as the essence of individuals.

### The Impact of Entrepreneurship Education and Entrepreneurial Efficacy on Entrepreneurial Intentions

In the research field of entrepreneurship, the initial focus is mainly on entrepreneurial intentions of entrepreneurs. For the research on entrepreneurial intentions, from the initial focus on individual factors to the later introduction of external environmental variables, the impacts of entrepreneurship education are gaining ever-increasing attention. Entrepreneurial intention is the antecedent variable of entrepreneurial behavior. Previous studies have shown that the level of entrepreneurship education has a significant effect on entrepreneurial intention. At present, the development of entrepreneurship education has become an important method to promote college students’ entrepreneurial activities. As a result, it has become a research hot spot. However, from the perspective of overall development, the current development of entrepreneurship education is still in the exploration stage. Therefore, it is necessary to conduct an overall investigation. On this basis, on the premise of cross-culture, the correlations among entrepreneurship education of Chinese American college students and the entrepreneurial intention of college entrepreneurs are explored and analyzed.

**FIGURE 1:**
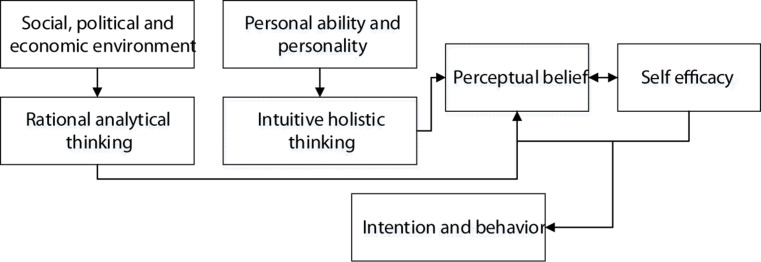
Model of entrepreneurial intention.

The manifestation of individual ability is inseparable from the entrepreneurial efficacy. Previous studies have shown that entrepreneurial efficacy has a satisfactory, predictive ability in terms of career. When there is a special entrepreneurial environment, the premise of individual entrepreneurial intentions is considered as a sense of entrepreneurial efficacy, which has a direct impact on the creation of entrepreneurial intentions and behaviors. Hence, entrepreneurial effectiveness has a greater impact on external environmental factors and individual psychological characteristics, which is a type of outcome variable. Entrepreneurial efficacy also has the ability to predict entrepreneurial intentions, which is a type of antecedent variable. On this basis, on the premise of cross-culture, whether the entrepreneurial efficacy of Chinese American college students has an intermediary role is explored and analyzed. Among them, the entrepreneurial intention model is shown in Figure 1 below.

### Design of Research

**FIGURE 2:**
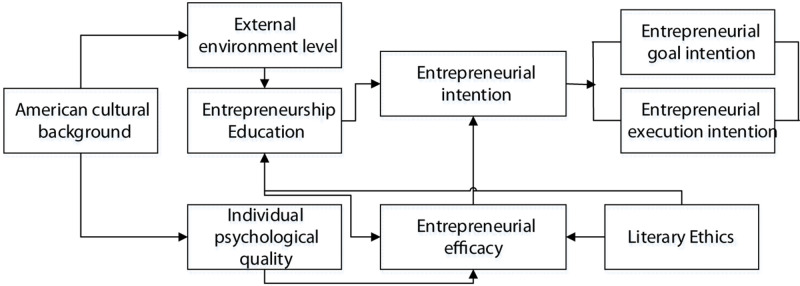
Model of correlation research.

In the research on entrepreneurship education, the above statements have shown that entrepreneurial behavior is complex and changeable, and there are many influencing factors. Among them, culture is one of the most important influencing factors. Given significant differences in history, culture, education, ideas, and lifestyle of different countries, the presentation of entrepreneurship education will also vary. In such a complex system, the entrepreneurial group of Chinese American college students will also face many ethical issues during the implementation of entrepreneurial behaviors. The maintenance and further improvement of their entrepreneurial efficacy will have a great impact on the success of entrepreneurship. In summary, entrepreneurial intention is the prerequisite for entrepreneurial behaviors and activities, and entrepreneurial efficacy is the antecedent variable of entrepreneurial intention. Entrepreneurship education occupies an important position in the creation of entrepreneurial intentional behaviors. Therefore, it is very necessary to discuss the impact of entrepreneurial intention. For this reason, in the study of Chinese American entrepreneurship education, the entrepreneurial intention is introduced. Based on the sense of entrepreneurial efficacy under psychological theory, the analysis is performed from the perspective of literary ethics. The establishment of the correlation research model is shown in Figure 2. The research hypotheses are presented in Table 1.

**TABLE 1:** Research hypotheses.

**Hypothesis number**	**Concrete content**
Hypothesis 1	There is a significant correlation between entrepreneurship education, entrepreneurial efficacy, and entrepreneurial intention of Chinese American college students.
Hypothesis 2	The entrepreneurial efficacy of Chinese American college students plays an intermediary role between entrepreneurial goal intention and entrepreneurial execution intention.
Hypothesis 3	The sense of entrepreneurial efficacy of Chinese American college students plays an intermediary role between entrepreneurship education and entrepreneurial intention.
Hypothesis 4	Gender and family entrepreneurial behavior of Chinese American college students play a moderating role in the structural model of entrepreneurial efficacy.

### Selection of Research Samples and Scale Tools

Among the studies in the cross-cultural field, the questionnaire survey method has the widest scope of application and is less affected by external factors (Kim et al., 2018). Considering the study of entrepreneurship education of Chinese American college students, the data were collected and analyzed by means of questionnaire survey. For the design of the questionnaire, a team of five Chinese American master’s graduates from the Department of Psychology of a certain American university revised the questionnaire accordingly. After many discussions, the final version of this questionnaire was determined. The questionnaires were distributed online. Research participants completed the questionnaire directly through Survey Monkey, and the feedback information of the questionnaire could be received in time. Considering the level of entrepreneurship education, the selected research samples include Chinese American college students who were over 18 years old and were educated in the United States. Before the results of the questionnaire survey were analyzed, the invalid questionnaires contained in the questionnaire were eliminated. The judgment criteria for the invalid questionnaires include these conditions: the questionnaire has many blanks, the question options are highly repeated, and the basic personal information is missing.

The entrepreneurial intention scale includes a total of 12 questions, and the evaluation method is 7-point scoring (Baldegger et al., 2017). For entrepreneurship education, the revised entrepreneurship education status scale was selected (Qu et al., 2017), which contained a total of seven questions, and the evaluation method also used the 7-point scoring method. For entrepreneurial efficacy, the Entrepreneurial Self-Efficacy Scale was chosen (Ayse and Turkan, 2018), which included a total of five dimensions and five questions, and the evaluation method used the 5-point scoring method. Two indicators, reliability and structural validity, were selected to test the reliability of the research variable scale. The reliability test was evaluated by Cronbach’s Alpha reliability coefficient, and the validity of the structural validity was tested by factor analysis method (Heuvelman et al., 2018). The test factors involved included Chi-square degree of freedom (χ^2^/df), root mean square error approximate (RMSEA), normed fitting index (NFI), satisfaction, of fit index (GFI), comparison fit index (CFI), and incremental fit index (IFI). For the statistical analysis of data, the SPSS 26.0 and AMOS 26.0 are utilized. Then, the multi-factor analysis of variance and regression analysis methods were used (Bauernhofer et al., 2018; Lu et al., 2019). Combined with the construction of structural equation models and based on the perspective of literary ethics, the correlations among entrepreneurial intention, entrepreneurship education, and entrepreneurial efficacy were explored. The measurement scale of each research variable is shown in Table 2.

**TABLE 2:** Measurement scale of each research variable.

**Research variable dimension**	**Measurement problems**
Entrepreneurial intention	1.1 Do my best to set up and run my own company.
	1.2 My career goal is to be an entrepreneur.
	1.3 I’m interested in starting a company.
	1.4 I will start my own company in 5 years.
	1.5 I wish I could be the boss.
	1.6 When I feel that my economic situation is not optimistic, I will choose to start a business.
	1.7 When I’m not interested in what I’m doing, I start a business.
	1.8 If I can’t find a satisfactory job when I graduate, I will start my own business.
	1.9 When I am faced with great work pressure, I will start a business.
	1.10 When I have studied the relevant entrepreneurship courses, I will start my own business.
	1.11 When I find the right partner, I will start a business.
	1.12 Setting up my own company is within the scope of my plan.
Entrepreneurship education	2.1 The school has special entrepreneurship education courses.
	2.2 The school has an entrepreneurial fund.
	2.3 The school has a strong atmosphere of entrepreneurship and innovation.
	2.4 Most of the lecturers in the school are successful entrepreneurs.
	2.5 The school is equipped with a professional team of entrepreneurship education teachers.
	2.6 The setting of university courses increases students’ entrepreneurial skills.
	2.7 What I learned at school is ready for Entrepreneurship.
Entrepreneurial efficacy	3.1 Go all out to deal with the problem
	3.2 Believe in my social skills to cope with any situation.
	3.3 Be able to consider and solve problems from multiple perspectives.
	3.4 As long as we are diligent and practical, we can solve many problems.
	3.5 It’s easy in any situation.

## Results

### Questionnaire Statistics

The demographic characteristics of the selected research samples are shown in Table 3. A total of 600 questionnaires were distributed, 570 of which were finally recovered. After invalid questionnaires were removed from the recovered questionnaires, the number of valid questionnaires was 550, and the validity rate was 96.49%. As shown in Table 3, the proportion of boys in the research samples is 48.7%, and the proportion of girls is 51.3%. In addition, for the majors of research samples, the proportion of science and engineering majors is 45.3%, the proportion of literature and history majors is 7.2%, the proportion of economics and management majors is 22.2%, and the proportion of social science majors is 25.3%, of which the proportion of undergraduate students is larger, which accounts for 90.2%.

**TABLE 3:** Demographic characteristics of the research samples.

**Influence parameter**	**Classification and composition**	**Percentage (%)**
Gender	Male	51.3
	Female	48.7
Age	Over 25 years old	46
	21–25 years old	47
	18–20 years old	7
Educational background	Master	9.8
	Undergraduate	90.2
Major	Science and Engineering	45.3
	Literature and History	7.2
	Economic Management	22.2
	Social Sciences	25.3
Family Entrepreneurship	Yes	25
	No	75

### Scale Reliability and Validity Test Results

**FIGURE 3:**
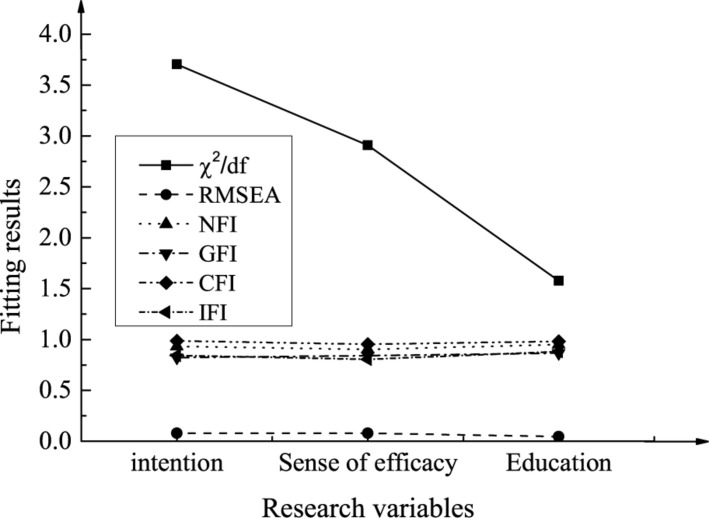
Structural validity test results.

The reliability tests of scale tools for the entrepreneurial intention, entrepreneurial efficacy, and the entrepreneurship education of Chinese American college students are shown in Table 4 and Figure 3 below.

**TABLE 4:** Reliability test.

**Research variables**	**Entrepreneurial intention**	**Entrepreneurial efficacy**	**Entrepreneurship education**
Cronbach’s alpha	0.90	0.87	0.92

As shown in Table 4, the Cronbach’s Alpha reliability coefficient corresponding to entrepreneurial intention is 0.90, the Cronbach’s Alpha reliability coefficient corresponding to entrepreneurial efficacy is 0.87, and the Cronbach’s Alpha reliability coefficient corresponding to entrepreneurship education is 0.92. The reliability coefficients of the variables are all above 0.80, indicating that the reliability of the scale is satisfactory. As shown in Figure 3, for entrepreneurial intentions, χ^2^/df = 3.703, RMSEA = 0.078, NFI = 0.933, GFI = 0.823, CFI = 0.986, IFI = 0.844. For entrepreneurial efficacy, χ^2^/df = 2.908, RMSEA = 0.078, NFI = 0.902, GFI = 0.842, CFI = 0.953, IFI = 0.804. For entrepreneurship education, χ^2^/df = 1.577, RMSEA = 0.045, NFI = 0.951, GFI = 0.866, CFI = 0.982, IFI = 0.883. Therefore, the structural validity of the scale is satisfactory.

### Descriptive Statistical Analysis

**FIGURE 4:**
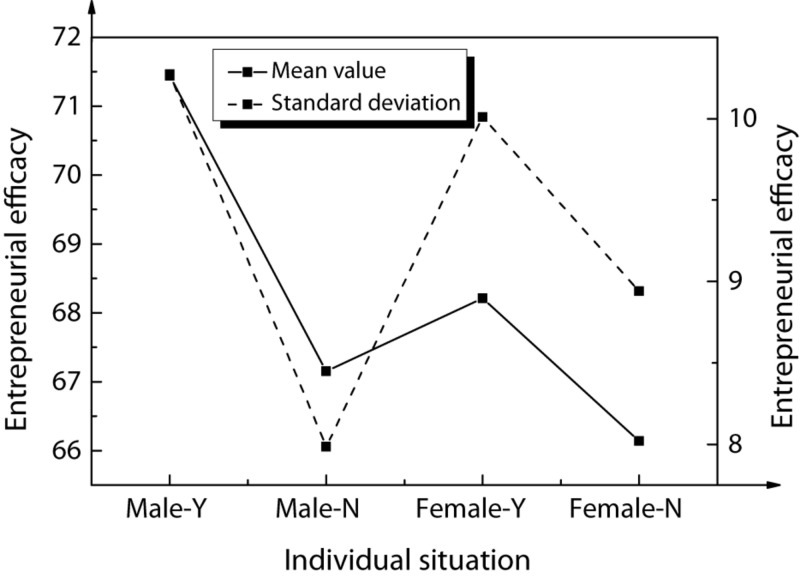
Results of variance analysis.

The entrepreneurial efficacy was taken as the dependent variable. Then, the gender of Chinese American college students and the entrepreneurial behaviors of their family members were taken as independent variables. The corresponding variance analysis results are shown in Figure 4 below.

In the above figure, Male-Y represents a male college student, and his family has entrepreneurial behaviors. The rest may be deduced by analogy.

**FIGURE 5:**
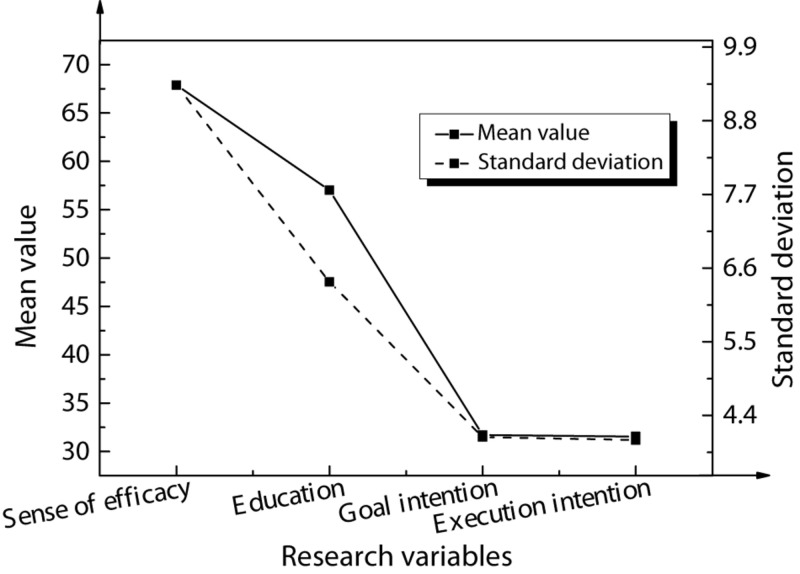
Descriptive statistical analysis.

Analysis of data in the figure shows that in terms of entrepreneurial efficacy, Chinese American college students have significant differences in family entrepreneurial behaviors. The descriptive statistics and correlation analysis results of the corresponding variables of the research samples are shown in Table 5 and Figure 5.

**TABLE 5:** Correlation analysis.

	**Entrepreneurial efficacy**	**Entrepreneurship education**	**Entrepreneurial goal intention**	**Entrepreneurial execution intention**
Entrepreneurial efficacy	1			
Entrepreneurship education	0.432**	1		
Entrepreneurial goal intention	0.385**	0.436**	1	
Entrepreneurial execution intention	0.213**	0.369**	0.552**	1

**p < 0.01.

As shown in Table 5, there is a significant positive correlation between the two dimensions of entrepreneurial intention, i.e., entrepreneurial goal intention and entrepreneurial execution intention. There is significant positive correlations among entrepreneurial goal intention, entrepreneurial efficacy, and entrepreneurship education. There are significant positive correlations among entrepreneurial intention, entrepreneurial efficacy, and entrepreneurship education. At the same time, there are also significant positive correlations between entrepreneurial efficacy and entrepreneurial goal intention, and entrepreneurial execution intention and entrepreneurship education.

### Test of the Intermediary Role of Entrepreneurial Efficacy

The fit index of the structural model of the correlations among entrepreneurial intentions, entrepreneurship education, and entrepreneurial efficacy of Chinese American college students is shown in Table 6.

**TABLE 6:** Fit index of correlation structure model.

**Confirmatory factor**	**χ^2^/df**	**RMSEA**	**NFI**	**GFI**	**CFI**	**IFI**
Fitting results	2.454	0.079	0.923	0.931	0.912	0.912

As shown in Table 6, the correlation structure model has a satisfactory, fitting effect, and the path coefficient among the entrepreneurial efficacy, entrepreneurial goal intention, and entrepreneurial execution intention is significant, which shows that the entrepreneurial efficacy has a partially intermediary effect for the entrepreneurial goal intention and entrepreneurial execution intention. Furthermore, the path coefficients between entrepreneurship education and entrepreneurial goal intention, and entrepreneurial execution intention and entrepreneurial efficacy, are significant, which reveals the correlation between entrepreneurship education and entrepreneurial intention of entrepreneurial efficacy.

**FIGURE 6:**
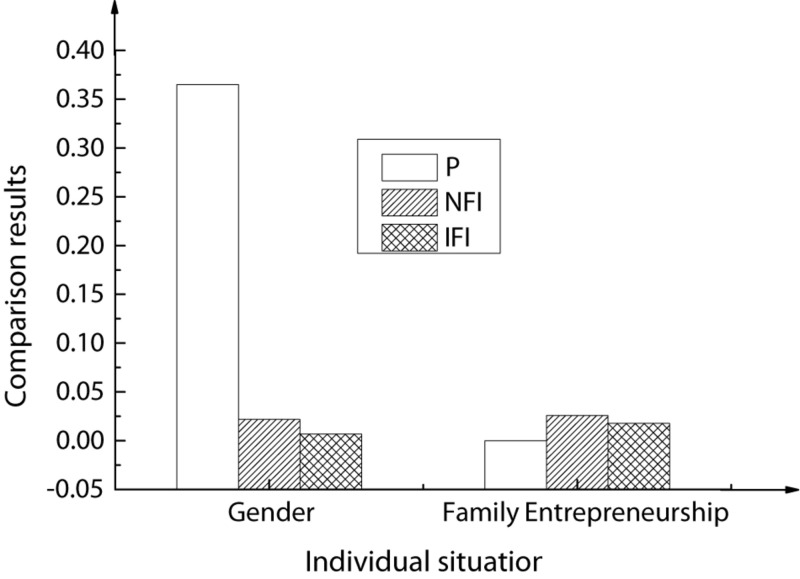
Cross-group comparative analysis based on gender and family entrepreneurship background.

**FIGURE 7:**
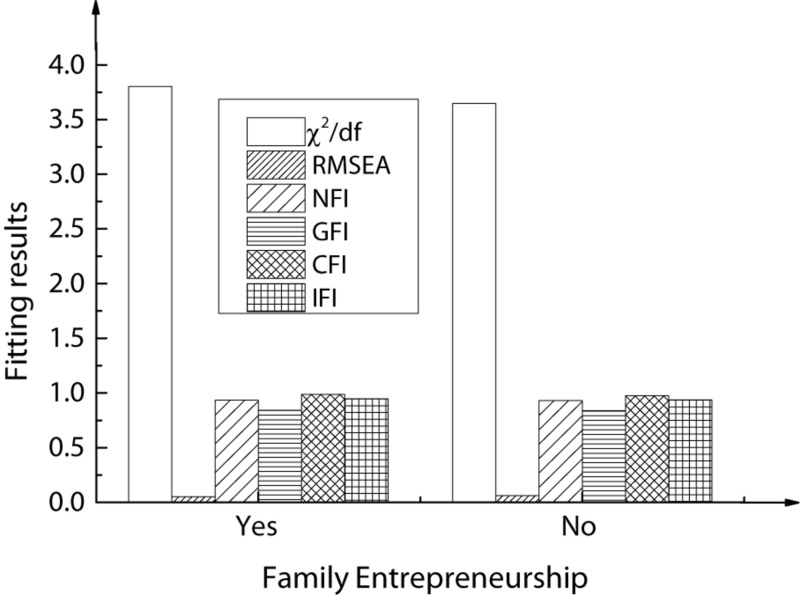
Model fit index of family members’ entrepreneurial behavior.

For Chinese American college students of different genders and family entrepreneurial behaviors, the corresponding cross-group comparative analysis results are shown in Figures 6, 7.

As shown in the above figures, in the restriction model, when the critical ratio P is greater than 0.05, the corresponding parameter changes are not significant. Accordingly, it is speculated that for Chinese American college students, individual genders have a significant regulatory impact on the correlations among entrepreneurship education, entrepreneurial efficacy, and entrepreneurial intentions. When the critical ratio P is smaller than 0.001, for family entrepreneurial behaviors, the changes in corresponding parameters are not very significant. Therefore, for Chinese American college students, family members’ entrepreneurial behaviors have a significant regulatory impact on the correlations among entrepreneurship education, entrepreneurial efficacy, and entrepreneurial intentions.

## Discussion

During the implementation of entrepreneurship education for college students, entrepreneurial efficacy is an expression of individual characteristics based on psychological theory. Entrepreneurial intention behaviors, including entrepreneurial goal intentions and entrepreneurial execution intentions, are greatly affected by entrepreneurship education and entrepreneurial efficacy, in which entrepreneurial efficacy is the antecedent variable and has a predictive effect on entrepreneurial intention behaviors. The enhancement of entrepreneurial efficiency has the role of promoting the improvement of college students’ comprehensive quality, the cultivation of entrepreneurial innovation, the establishment of confident personality, and the persistence of perceptual belief. It plays an important role in the development of college students’ entrepreneurial ethics. The results show that both different genders and different family entrepreneurial behaviors have a significantly positive impact on entrepreneurial efficacy of college students. Considering that entrepreneurial efficacy will not change with cultural differences or different life backgrounds, there will also be no significant differences in the context of transnational or cross-cultural backgrounds. Through analysis, it is found that Chinese American college students whose family members have entrepreneurial behaviors have a significantly higher entrepreneurial efficiency than students without family members’ entrepreneurial behaviors. Therefore, family members’ entrepreneurial behaviors are a family background factor of entrepreneurial ethics, which is of great significance to the improvement of entrepreneurial efficiency of college entrepreneurs.

The descriptive statistical analysis results of the research variables show significant correlations between the entrepreneurial efficacy and entrepreneurship education, and the entrepreneurial goal intentions and entrepreneurial execution intentions of Chinese American college students. The proposed Hypothesis 1 is verified, and it is reasonable. Therefore, scholars have found that for entrepreneurial intentions, entrepreneurial efficacy is an important predictor variable. Entrepreneurial efficacy has a guiding role in the development of entrepreneurial intentions (Simone et al., 2017). In addition, for the possibility of entrepreneurial efficacy as an intermediary variable in the field of entrepreneurship research, previous studies have explored it (Klompstra et al., 2017). By constructing a structure model of the correlations among entrepreneurship education, entrepreneurial efficacy, and entrepreneurial intention of Chinese American college students, it is found that the entrepreneurial efficacy of college students has a significantly positive predictive effect on entrepreneurial intention, which is consistent with the conclusions drawn by previous studies (Barba-Sánchez and Atienza-Sahuquillo, 2018). Entrepreneurial efficacy has a partial intermediary role between entrepreneurial goal intentions and entrepreneurial execution intentions. The correlation structure model has found that the path coefficient of entrepreneurship education to entrepreneurial intention and entrepreneurial efficacy of Chinese American college students is significant. Entrepreneurial efficacy has a partially mediating effect on the correspondence between entrepreneurship education and entrepreneurial intention. The level of entrepreneurial efficacy developed through self-efficacy has a close correlation with the level of education received by the individual. Entrepreneurial intentions can be used to predict the perception of entrepreneurial efficacy, which verifies that entrepreneurial efficacy plays an intermediary role between entrepreneurship intention and entrepreneurship education. This also validates the rationality of the proposed Hypothesis 2 and Hypothesis 3.

For the role of gender and family entrepreneurial behaviors in the correlation structure model, the results show that the two have a regulatory effect on entrepreneurial efficacy. This verifies Hypothesis 4 and illustrates the rationality of this hypothesis. In general, entrepreneurial efficacy plays an intermediary role between entrepreneurial ethics and entrepreneurial intention. However, this impact is affected by the gender differences and family entrepreneurial behaviors of Chinese American college students. Thus, the development of individual entrepreneurs is the result of the interactions among a variety of factors. Therefore, college entrepreneurs should seize the opportunities of ethical strategies and fully utilize their advantages to achieve the purpose of successful entrepreneurship (Wu et al., 2019). When college students choose to start their businesses, no matter what stage they are in, it is very important for them to improve their personal qualities and avoid ethical and moral anomie behaviors. In addition, the improvement of entrepreneurial efficiency is an important aspect.

## Conclusion

In this study, the Chinese American college students were chosen from American colleges and universities as the research samples. Based on the perspective of psychology and literary ethics, it is found that there are significant correlations among entrepreneurial efficacy, entrepreneurial intention, and entrepreneurship education. Among them, entrepreneurial efficacy has a partially intermediary role, and individual gender and family background have a regulating impact on entrepreneurial behavior. However, due to various factors, the selection of research samples is not comprehensive enough in terms of grades and majors. In addition, due to the cross-cultural factors, cultural differences are inevitable. In the future, therefore, more comprehensive research samples need to be introduced, and multiple influencing factors including culture should be analyzed, so as to further deepen the proposed topic.

## Data Availability Statement

The raw data supporting the conclusions of this article will be made available by the authors, without undue reservation, to any qualified researcher.

## Ethics Statement

The studies involving human participants were reviewed and approved by the Chongqing Jiaotong University Ethics Committee. The patients/participants provided their written informed consent to participate in this study.

## Author Contributions

All authors listed have made a substantial, direct and intellectual contribution to the work, and approved it for publication.

## Conflict of Interest

The authors declare that the research was conducted in the absence of any commercial or financial relationships that could be construed as a potential conflict of interest.
